# A generalization of Nash's theorem with higher-order functionals

**DOI:** 10.1098/rspa.2013.0041

**Published:** 2013-06-08

**Authors:** Julian Hedges

**Affiliations:** School of Electronic Engineering and Computer Science, Queen Mary University of London, London E1 4NS, UK

**Keywords:** selection function, normal-form game, Nash equilibrium, higher-order function

## Abstract

The recent theory of sequential games and selection functions by Escardó & Oliva is extended to games in which players move simultaneously. The Nash existence theorem for mixed-strategy equilibria of finite games is generalized to games defined by selection functions. A normal form construction is given, which generalizes the game-theoretic normal form, and its soundness is proved. Minimax strategies also generalize to the new class of games, and are computed by the Berardi–Bezem–Coquand functional, studied in proof theory as an interpretation of the axiom of countable choice.

## 1. Introduction

The notion of *optimization* is common to many areas of applied mathematics, such as game theory and linear and nonlinear programming. Typically, we have a set *X* of *choices* and a function *p* mapping each *x*∈*X* to a real number *p*(*x*), which we might call the *value* or *cost* of *x*. From this we can define a natural notion of *optimality*: a point 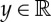 is optimal just if *y*≥*p*(*x*), for all *x*∈*X* and *y*=*p*(*x*_0_) for some *x*_0_∈*X*. We usually refer to *y* by a notation such as

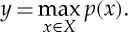
The point *x*_0_ is also interesting: it is a point at which *p* *attains* its optimal value, and we refer to it as

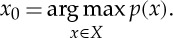
(Of course, while *y* is guaranteed to be unique when it exists, *x*_0_ is not necessarily unique; we only require that arg max chooses some value for *x*_0_.) These notations are connected by the equation *y*=*p*(*x*_0_), or

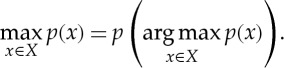


Let *X* be a finite set, so that 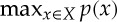 is well defined for all functions 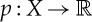. We can now define a function by

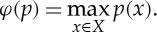
*φ* has range 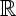, and its domain is the function set 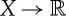, that is, the set of all functions with domain *X* and range 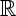. We, therefore, write

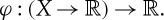
We call *φ* a *higher-order function* (or *functional*), that is, a function whose domain is itself a set of functions. We can also define

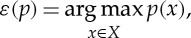
obtaining a higher-order function

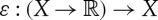
satisfying

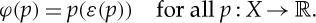


Using the concept of a higher-order function, we can make a large generalization of the properties of 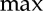 and arg max. For any sets *X* and *R*, a function *φ*:(*X*→*R*)→*R* will be called a *quantifier* and a function *ε*:(*X*→*R*)→*X* will be called a *selection function*. Intuitively, a quantifier is a rule for converting a function *p*:*X*→*R* into an element of *R* considered, in an abstract sense, to be the ‘most desirable’, and a selection function produces instead a value in *X* at which *p* takes its most desirable value. We say that *ε* *attains* *φ* just if *φ*(*p*)=*p*(*ε*(*p*)) for all *p*:*X*→*R*.


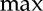
 and arg max are the prototypical examples of a quantifier and a selection function attaining it. A very different example is a fixed point operator *μ*:(*X*→*X*)→*X*, which has the property that *μ*(*p*) is always a fixed point of *p*, that is, *μ*(*p*)=*p*(*μ*(*p*)). Although this cannot be understood as a set-theoretic function, it is well defined in models of partial computable functionals, and there can be seen as both a quantifier and a selection function, attaining itself. Quantifiers where *R* is the set of truth-values appear naturally in logic, and include the classical quantifiers ∀ and ∃ (this is the reason for the name *quantifier*). A slightly more general notion of *multi-valued quantifier* is defined in the next section. These concepts were introduced and applied to the theory of sequential games by Escardó & Oliva [1] in a series of papers summarized in [1].

What is a game? Typically, some players take turns choosing between sets of legal moves, which may be constrained by previous players’ moves. The sequence of moves made by the players is called a *play* of the game. Usually, the rules of the game guarantee that every play terminates after a finite number of moves, and then uniquely determine which player has won the play.

In the theory of games as introduced by von Neumann & Morgenstern [2], the notion of a player winning a play is not used. Rather, for each player, the rules of the game define an *outcome function* mapping each play of the game to a real number called the *utility* of the play for that player. This generalization is important for applications of game theory to economics, where utility often represents profit. In the game played by two competing firms, for example, each firm is interested in maximizing its own profit, and does not care (in the short term, at least) how much profit its competitor makes. Of course, a firm's profits will be affected by the moves of its competitor, and vice versa. A central problem of game theory is to determine which moves each player should choose in order to maximize their utility. The theory of games as surveyed for example in [3] will be referred to as *classical game theory*.

Suppose, during the course of a play, some player must choose between some set *X* of moves. Taking the usual assumption of common knowledge of rationality (that is, the players play optimally, and they know that each other will play optimally, and so on) the future of the play after making each choice of *x*∈*X* is sufficiently well determined that a utility 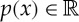 can be assigned to each *x*∈*X*. In classical game theory, a rational player will always choose arg max_*x*∈*X*_*p*(*x*). By replacing 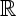 with an arbitrary set *R* and arg max with an arbitrary selection function *ε*:(*X*→*R*)→*X*, a rich theory of generalized games results, with deep connections to proof theory and theoretical computer science [4,5].

The games that have been described so far are the so-called *sequential* games. In the more usual language of classical game theory, this can be read as *non-branching extensive form games of perfect information*. However, there are games that cannot be described as a sequence of moves. These are the so-called *simultaneous* games or *games of imperfect information*. A well-known example is rock–paper–scissors; a more important example is the simultaneous pricing of goods by supermarkets. von Neumann and Morgenstern proved that every game can be described as a simultaneous game, called its *normal* or *strategic form*. The central idea of this proof is that players simultaneously choose *contingent strategies*, higher-order functions that choose the next move given the partial play up to that point, and so play the game on behalf of the player. In this paper, we consider a notion of simultaneous games that encompasses Escardó and Oliva's generalized sequential games in a similar way. Given the number of applications of generalized sequential games (for example to the technology of *program extraction*, or the development of provably correct software), it is hoped that similar applications will appear for generalized simultaneous games, although these are not investigated in this paper.

In §3, generalized simultaneous games and their appropriate notion of equilibrium are defined. In §4 a class of games, the so-called *multilinear games*, is defined, and it is proved that games of this kind always have an equilibrium (theorem 4.13). This is used in §5 to prove the key result of this paper (theorem 5.7), a natural generalization of Nash's theorem for the existence of mixed-strategy equilibria to games defined by arbitrary quantifiers. In §6, a mapping from sequential to simultaneous games is defined analogous to the normal form construction in the classical theory, and its soundness is proved (theorem 6.4). In §7, we show an interesting connection to proof theory, namely that the *binary Barardi–Bezem–Coquand functional* computes minimax strategies of games, a result that suggests a deeper connection between proof theory and generalized games.

## 2. Preliminaries

If *X* and *Y* are sets, then *X*→*Y* denotes the set of all functions with domain *X* and range *Y* (this is often denoted *Y* ^*X*^, a notation we avoid in order to avoid writing exponential towers for higher-order functions). Cartesian products of sets are denoted 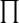 and bind tighter then →, so for example, 
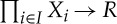
 means 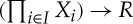. The *i*th coordinate projection of a tuple 
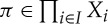
 is denoted *π*_*i*_.

The following piece of notation, for manipulating products, will be helpful. Let *I* be a set and let *X*_*i*_ be a set for each *i*∈*I*. If *x*∈*X*_*i*_ and 
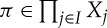
, then we define 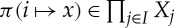 by

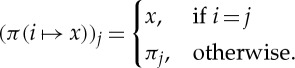


We make use of Church's *λ*-notation for describing functions anonymously. The function that might otherwise be written as *x*↦1+*x* will be denoted 
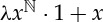
, where 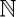 is the domain of the anonymous function. For example, we have 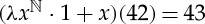. A variable bound by a *λ* need not appear under the scope of the *λ*, for example, *λx*^*X*^⋅42 is the constant function with the property that (*λx*^*X*^⋅42)(*x*′)=42, for all *x*′∈*X*.

A *quantifier* is a function *φ*∈*S*_*R*_(*X*) where 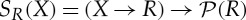 (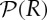 being the set of all subsets of *R*), a definition introduced in [1]. These ‘multi-valued’ quantifiers slightly generalize the single-valued quantifiers discussed in the previous section, by allowing zero or more distinct ‘optimal’ values in *R*. For example, we can interpret a fixed-point operator set-theoretically as a multi-valued quantifier, by letting *μ*(*p*) be the (possibly empty) set of *all* fixed points of the function *p*∈*X*→*X*.

The *domain* of a quantifier is

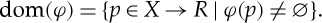
A quantifier with dom(*φ*)=*X*→*R* will be called *total*.

A *selection function* is a function *ε*∈*J*_*R*_(*X*), where *J*_*R*_(*X*)=(*X*→*R*)→*X*. Selection functions were first introduced in [6]. The quantifier *φ*∈*S*_*R*_(*X*) is *attained* by the selection function *ε*∈*J*_*R*_(*X*) just if

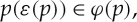
for all *p*∈dom(*φ*). This definition of attainment differs from Escardó and Oliva's, who require the condition to hold for all *p*∈*X*→*R*. For a total quantifier (which are considered in §5, and to which the main theorem applies), the two definitions coincide.

For example, if 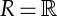 and *X* is a compact topological space, then the extreme value theorem (plus the axiom of choice) implies that the maximum quantifier

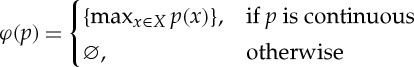
is attained. (Note that we need the axiom of choice to collect all the values into a single function.) A quantifier such as this whose values have cardinality at most 1 will be called *single-valued*. Because the definition of *φ* is clumsy, we can use a new notation for single-valued quantifiers, such as

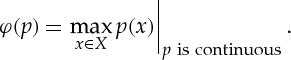


We assume some point-set topology as covered, for example, in [7] and elementary properties of topological vector spaces [8]. All topological vector spaces are assumed to be *T*_1_ throughout (this is no loss of generality because quotienting a topological vector space by the closure of {0} always yields a Hausdorff space). For reference, a subset *S* of a real vector space is called *convex* iff for all *x*,*y*∈*S* and *t*∈[0,1], we have *tx*+(1−*t*)*y*∈*S*.

In §4, we work with the class of *locally convex spaces*. The definition of a locally convex space is technical and not necessary for our purposes; beyond theorem 4.5 and lemma 4.6, we only need to know that every locally convex space is a topological vector space. Every normed vector space is locally convex; examples of locally convex spaces that are not normable include the spaces of smooth functions 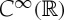 and 
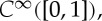
 and the space of real-valued sequences 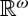 with convergence defined pointwise. Locally convex spaces are covered in detail in [8].

### (a) A note on foundations

It is possible to define generalized sequential games over models other than classical set theory. Indeed, as explained in [1], it is sometimes necessary to work in non-standard models, for example when considering unbounded sequential games (which are not considered in this paper). In particular, unbounded games are known to be well-behaved in the models of continuous functionals [9] and majorizable functionals [10]. The operation *J*_*R*_(*X*)=(*X*→*R*)→*X* is a (strong) monad and can be defined over any Cartesian closed category. Moreover, the closely related *K*_*R*_(*X*)=(*X*→*R*)→*R*, which contains the total single-valued quantifiers, is already well known from programming language theory, where it is called the *continuation monad*, introduced in the classic paper [11]. The definitions of generalized simultaneous game and generalized Nash equilibrium could be formalized in a more general setting, but the proofs in §4 use classical set theory in an essential way, so we find it easier to avoid foundational issues altogether and work entirely in classical set theory.

## 3. Generalized simultaneous games

In this section, we define the objects studied in this paper, namely *generalized simultaneous games* and *generalized Nash equilibria*. The definition of a generalized simultaneous game comes from the classical definition of a normal-form game, but with the maximizing behaviour of players replaced with a specified quantifier. For the general definition, we do not require the number of players to be finite. The related notion of *generalized sequential game* will be defined in §6.

**Definition 3.1 generalized simultaneous gamegeneralized simultaneous game**

A *generalized simultaneous game* (with multiple outcome spaces), denoted simply *game* when not ambiguous, is a tuple

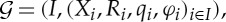
where *I* is a non-empty set of *players*, and for each *i*∈*I*,

— *X*_*i*_ is a non-empty set of *strategies* for player *i*;
— *R*_*i*_ is a set of *outcomes* for player *i*;
— *q*_*i*_∈*S*→*R*_*i*_ is the *outcome function* for player *i*, where 
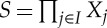
 is the *strategy space* of 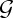; and
— *φ*_*i*_∈*S*_*R*_*i*__(*X*_*i*_) is the *quantifier* for player *i*.

We say that 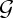 has a *single outcome space,* if the *R*_*i*_ are equal and the *q*_*i*_ are equal. In this case, 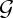 is determined by a tuple

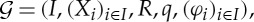
where *q*∈*S*→*R*.

An element *x*∈*X*_*i*_ is called a *strategy* for player *i* for 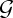. A tuple *π*∈*S* is called a *strategy profile* or *strategy* for 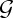. Throughout this paper the variables *π*, *σ* and *τ* will range over strategies of a game.

In general, we need games with multiple outcome spaces to study simultaneous games, and in particular, to recover the classical Nash theorem. However, normal forms of generalized sequential games will always have a single outcome space.

The appropriate notion of equilibrium of a generalized simultaneous game is called a *generalized Nash equilibrium*. Before making this definition, we first define some notation used throughout this paper. First, we define the family of *unilateral maps* 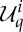, which are used as a shorthand notation but, when considered as a higher-order functions, are also natural and interesting in their own right.

**Definition 3.2 (unilateral map)**

Let *I* be a set, and for each *i*∈*I*, let *X*_*i*_ and *R*_*i*_ be sets. Let *q*=(*q*_*i*_)_*i*∈*I*_ be a family of maps such that each

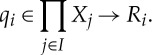
We define the *ith* *unilateral map*

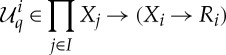
by

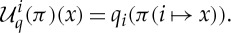

Thus, the *ith* unilateral map computes the outcomes of unilateral changes of strategy by the *ith* player in a game. Second, we associate to every quantifier a set called its *diagonal*.

**Definition 3.3 (diagonal of a quantifier)**

Let *φ*∈*S*_*R*_(*X*) be a quantifier. The *diagonal* of *φ* is

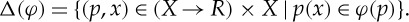

Now, the equilibria of a generalized simultaneous game can be defined in a very compact and (as will be seen) useful way.

**Definition 3.4 (generalized Nash equilibrium)**

Let 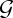 be a game with strategy space *S*. We define the *best response correspondence* 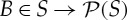 of 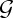 by

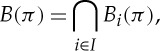
where the 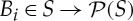 are defined by

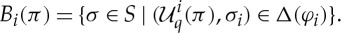

A *generalized Nash equilibrium* of 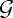 is a fixed point of *B*, that is, a strategy profile *π* such that *π*∈*B*(*π*).

Unpacking this definition, we see that *π* is a generalized Nash equilibrium of 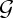 iff for each *i*∈*I*, we have

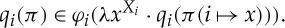
When *X*_*i*_ is compact, *q*_*i*_ is continuous and *φ*_*i*_ is the quantifier

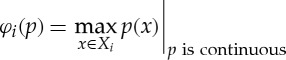
this reduces to

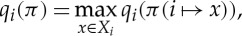
which is the usual definition of a Nash equilibrium.

## 4. Multilinear games

Now we define a large family of games, called the *multilinear games*, that are guaranteed to have a generalized Nash equilibrium. The structure of the argument is the same as that in [12], but given in more generality to deal with more general quantifiers. This section can be seen as a series of lemmas that are eventually used to prove theorem 5.7 (the generalization of Nash's theorem) in the next section. In fact the machinery developed in this section is stronger than necessary to prove theorem 5.7: it should also be possible using multilinear games to prove an analogous generalization of Glicksberg's theorem [13], which in turn generalizes Nash's theorem from finite sets of strategies to compact spaces of strategies, with continuous outcome functions.

**Definition 4.1 (closed graph property)**

Let *X* and *Y* be topological spaces and 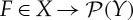. We say that *F* has *closed graph* iff

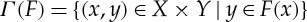
is closed with respect to the product topology.

The closed graph property is a form of continuity for functions whose range is a set of subsets of a topological space.

In order to guarantee that a generalized simultaneous game will have an equilibrium, we need to impose closed graph properties on the quantifiers. However, the domain of a quantifier is a function set, which in general has no unique natural topology. The least we need is that the unilateral maps are continuous, and so for this reason, we define the *unilateral topology*.

**Definition 4.2 (unilateral topology)**

For each *i*∈*I*, let *X*_*i*_ and *R*_*i*_ be topological spaces with *q*_*i*_∈*X*_*i*_→*R*_*i*_ continuous. The *unilateral topology* on *X*_*i*_→*R*_*i*_ is the final topology with respect to the singleton family 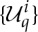, that is, it is the largest topology with respect to which 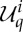 is continuous. A function which is continuous with respect to the unilateral topology will be called *unilaterally continuous*, and a function that has closed graph with respect to the unilateral topology has *unilaterally closed graph*.

Another possible topology on *X*_*i*_→*R*_*i*_ which will be useful is the topology of pointwise convergence. Most of this paper could be formulated using only pointwise convergence, except for an interesting example at the end of this section that needs a finer topology, namely uniform convergence.

**Lemma 4.3**

*The unilateral topology is finer than the topology of pointwise convergence*.

**Proof.**

It must be proved that 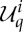 is continuous with respect to the topology of pointwise convergence. Let *π*_*j*_→*π* be a convergent sequence in 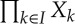, and let *x*∈*X*_*i*_. We have

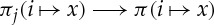
in the product topology, so

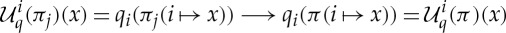
because *q*_*i*_ is continuous. Therefore, 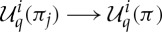 pointwise, as required. □

Now, we can give the definition of a multilinear game. This definition essentially contains the least assumptions needed for Nash's proof.

**Definition 4.4 (multilinear game)**

A game 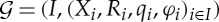 is called *multilinear* iff

(i) each *X*_*i*_ is a compact and convex subset of a given locally convex space *V*_*i*_ over 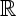;
(ii) each *R*_*i*_ is a topological vector space over 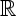;
(iii) each *q*_*i*_ extends to a continuous multilinear map

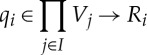
(that is, *q*_*i*_ is linear with respect to each *V*_*j*_ separately); and
(iv) each *φ*_*i*_ has unilaterally closed graph, *φ*_*i*_(*p*) is closed and convex for all *p*∈*X*_*i*_→*R*_*i*_, and 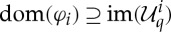.

(Note that because *q*_*i*_ is continuous and multilinear, to satisfy the last condition it suffices that 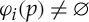 whenever *p* is continuous and linear. Note also that if *φ*_*i*_ is single-valued, then each *φ*_*i*_(*p*) is automatically closed and convex.)

The idea of the existence proof is to reduce to the following fixed point theorem.

**Theorem 4.5 (Kakutani–Fan–Glicksberg fixed point theorem [14,13])**

*Let S be a non-empty, compact and convex subset of a locally convex space over* 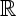
*. Let* 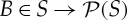 *have closed graph and let B(π) be non-empty, closed and convex for all π∈S. Then B has a fixed point, that is, there is a point π∈S such that π∈B(π).*

We will need to use the fact that locally convex spaces are closed under arbitrary products.

**Lemma 4.6**

*Let* {*V*_*i*_}_*i*∈*I*_ *be a family of locally convex spaces over a field* *K*. *Then* 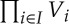 *has the structure of a locally convex space over* *K* *whose topology is the product topology*.

Much of the usefulness of multilinear games comes down to the fact that their unilateral maps are well-behaved.

**Lemma 4.7**

*Let* 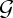 *be a multilinear game with strategy space* *S*. *Then each* 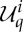 *is a continuous function*

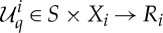
(*under the Curry bijection* *A*→(*B*→*C*)≅*A*×*B*→*C*) *and for each* *π*∈*S* *the map* 
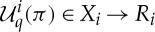
 *is linear*.

**Proof.**

By the continuity and multilinearity of the *q*_*i*_. □

Lemmas 4.8–4.12 form the core of the proof, establishing the hypotheses of the Kakutani–Fan–Glicksberg theorem.

**Lemma 4.8**

*Let* 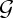 *be a multilinear game. Then the strategy space of* 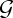 *is a non-empty, compact and convex subset of a locally convex space*.

**Proof.**

The strategy space is

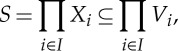
where the larger space is locally convex by lemma 4.6.

*S* is non-empty by the axiom of choice and compact by Tychonoff's theorem. Convexity is also inherited by the product, since for each *i*∈*I* we have

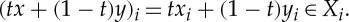
 □

**Lemma 4.9**

*Let* 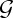 *be a multilinear game with best response correspondence* *B* *such that each quantifier* *φ*_*i*_ *is attained by a selection function* *ε*_*i*_. *Then* *B*(*π*) *is non-empty for all* *π*.

**Proof.**

Let *S* be the strategy space of 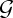. Given *π*∈*S*, we define *σ*∈*S* to have *ith* component

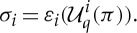
Because *ε*_*i*_ attains *φ*_*i*_ and 
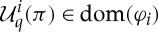
 (definition 4.4, point (iv)), we have

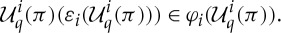
Therefore,

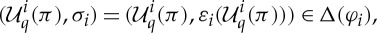
so *σ*∈*B*(*π*), as required. □

**Lemma 4.10**

*Let* 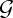 *be a multilinear game with best response correspondence* *B*. *Then* *B*(*π*) *is closed for all* *π*.

**Proof.**

It suffices to prove that each factor

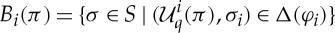
is closed. Let *σ*_*j*_→*σ* be a convergent sequence in *B*_*i*_(*π*). For each *i*, we have *σ*_*j*,*i*_→*σ*_*i*_, so

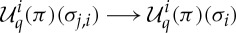
by the continuity of 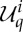. We also have that each

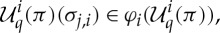
and the right-hand side is closed (definition 4.4, point (iv)), therefore,

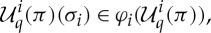
that is

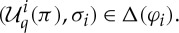

**Lemma 4.11**

*Let* 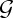 *be a multilinear game with best response correspondence* *B*. *Then* *B*(*π*) *is convex for all* *π*.

**Proof.**

Suppose *σ*,*τ*∈*B*(*π*) and *t*∈[0,1]. Let *i*∈*I*. By definition we have

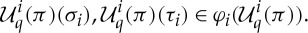
By the linearity of 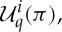 we have

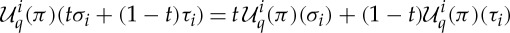
Because the *φ*_*i*_(*p*) are convex (definition 4.4, point (iv)), we have

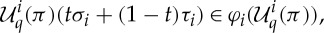
that is

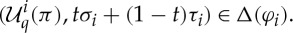
Therefore,

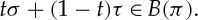
 □

**Lemma 4.12**

*Let* 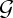 *be a multilinear game with best response correspondence* *B*. *Then* *B* *has closed graph*.

**Proof.**

Note that

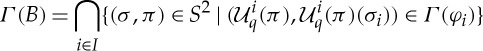
and so it suffices to prove these factors closed. Let (*σ*_*j*_,*π*_*j*_)→(*σ*,*π*) be a convergent sequence in the *ith* factor. By the continuity of 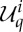,

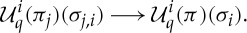

Because 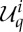 is also unilaterally continuous as a map *S*→(*X*_*i*_→*R*_*i*_), we have 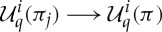 unilaterally. Therefore, we have a convergent sequence

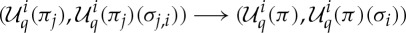
in the graph *Γ*(*φ*_*i*_), which is closed (definition 4.4, point (iv)). □

**Theorem 4.13 (existence theorem for multilinear games)**

*Let* 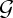 *be a multilinear game such that each quantifier is attained by a selection function. Then* 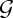 *has a generalized Nash equilibrium.*

**Proof.**

Let *B* be the best response correspondence of 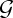. By lemmas 4.8–4.12 and the Kakutani–Fan–Glicksberg fixed point theorem, *B* has a fixed point. □

Examples of multilinear games as mixed extensions of finite games are given in the next section. Another interesting example is given by integration. Let *X*_*i*_=[0,1], 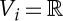 and 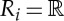, and let *L*(*X*_*i*_) be the set of all Lebesgue-integrable functions 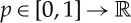 with

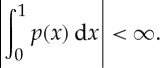
Define a single-valued quantifier 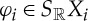 by

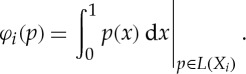
Using the mean value theorem (and the axiom of choice), we can prove the existence of a selection function attaining *φ*_*i*_: for all *p*∈*L*(*X*_*i*_) there exists *ε*_*i*_(*p*)∈*X*_*i*_ such that

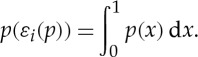
This highly non-constructive selection function was briefly introduced as an example in [6].

We let *I* be finite and let the other *X*_*j*_ be normed, so the strategy space is normed, and we can work with the *ε*−*δ* definitions of uniform convergence and continuity.

**Lemma 4.14**

*If q*_*i*_ *is uniformly continuous and π*_*j*_→*π* *then* 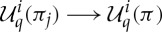 *uniformly*.

**Proof.**

We have that *q*_*i*_ is uniformly continuous, that is
4.1
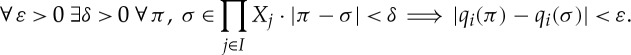
We also have *π*_*j*_→*π*, that is
4.2
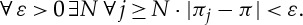
We want to prove that 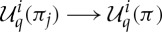 uniformly, that is

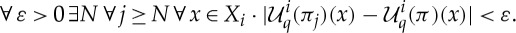

Let *ε*>0. By (4.1), we have *δ*>0 with the given property. We take *ε* in (4.2) to be this *δ*, obtaining *N*. Let *j*≥*N*, therefore,

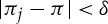
by (4.2). Let *x*∈*X*. The crucial observation is that *π*_*j*_(*i*↦*x*) behaves like *π*_*j*_ but is constant in its *ith* coordinate. That is, we have

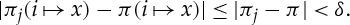

Now, we take *π*, *σ* in (4.1) to be *π*_*j*_(*i*↦*x*) and *π*(*i*↦*x*). We have already proved the antecedent in (4.1), therefore

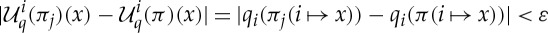
as required. □

We have proved that the unilateral topology is finer than the topology of uniform convergence.

**Lemma 4.15**

*φ*_*i*_ *is unilaterally continuous*.

**Proof.**

Suppose we have *p*_*j*_→*p* uniformly in *L*(*X*_*i*_). Because the convergence of the integrands is uniform, we can apply the uniform convergence theorem to get

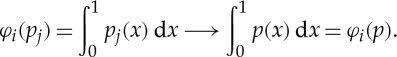
Because the unilateral topology is finer than the topology of uniform convergence, we are done. □

In the one-player game defined by the integration quantifier with outcome function *q*, the unique value of *q*(*x*) when *x* is an equilibrium strategy, which can be called the *expected outcome* of the game, is simply

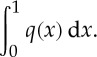
In the two-player game with both quantifiers integrals, a generalized Nash equilibrium (*a*,*b*) satisfies

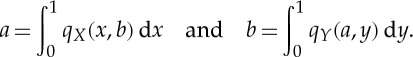
Because 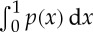 is the *average* value of *p*, this is a game where players are trying to gain the average outcome rather than the maximum. The existence of a Nash equilibrium in this case can be more directly proved by applying the Brouwer fixed point theorem to the mapping

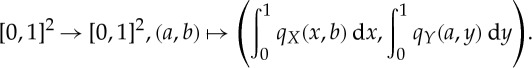


## 5. Finite games

In this section, we apply the existence theorem for multilinear games to prove a suitable generalization of Nash's theorem. The classical version of Nash's theorem guarantees that every finite game (that is, a classical game in which each player has finitely many strategies) has a *mixed strategy Nash equilibrium*.

The notion of mixed strategies means that we consider probability distributions over ordinary strategies (referred to as *pure strategies* for clarity). The outcome functions also need to be replaced by *expected outcome* functions. However, the discussion of probability distributions can be avoided by treating them as geometric objects, namely *simplices*. This approach also makes it clearer how quantifiers and selection functions must be modified when passing to mixed strategies. A probabilistic interpretation of the resulting theorem is possible, but is avoided in this paper.

**Definition 5.1 (finite game)**

A game 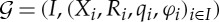 is called *finite* iff

— *I* is finite;
— each *X*_*i*_ is finite;
— each *R*_*i*_ is a topological vector space over 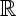; and
— each *φ*_*i*_ is total, has closed graph with respect to the topology of pointwise convergence (viewing the *X*_*i*_ as discrete topological spaces), and *φ*_*i*_(*p*) is closed and convex for all *p*∈*X*_*i*_→*R*_*i*_.

Note that restricting to pointwise convergence is no loss of generality here because the *X*_*i*_ are finite.

The set of probability distributions over a finite set can be seen as a geometric object called a *standard simplex*. In two and three dimensions these can be easily visualized as a line segment and an equilateral triangle; the next simplex *Δ*_4_ is a tetrahedron seen as a subset of 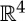.

**Definition 5.2 standard simplexstandard simplex**

The *nth* *standard simplex* is the set

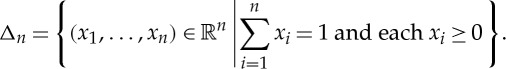

**Definition 5.3 (mixed extension)**

Let 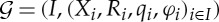 be a finite game with strategy space *S*. We define a game

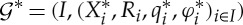
called the *mixed extension* of 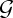 as follows. Player *i* has move set

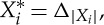
which, assuming a fixed enumeration of *X*_*i*_, can be seen as a simplex with vertices labelled by player *i*'s pure strategies (moreover, the coordinates of points in 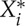 can be seen as being labelled by player *i*'s pure strategies). Player *i*'s outcome function is given by the formula

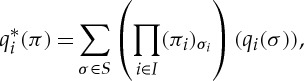
which computes the expected value of *q*_*i*_. The mixed strategy *π* is a tuple whose *ith* component *π*_*i*_ is a point in the simplex 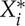, and so (*π*_*i*_)_*σ*_*i*__ is the coordinate of *π*_*i*_ labelled by the pure strategy *σ*_*i*_. 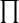 here represents ordinary multiplication of real numbers, so 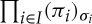 is a real number. 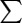 represents addition of vectors in *R*_*i*_, and the multiplication of the terms in brackets is multiplication of the vector *q*_*i*_(*σ*) by the real scalar 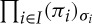, and so the entire formula is simply a linear combination in *R*_*i*_. (Note that the finiteness of *I* is used implicitly here: for the strategy space *S* to be finite it is necessary that *I* be finite, except in trivial cases when all but finitely many *X*_*i*_ are singletons.) Finally, player *i*'s quantifier is given by

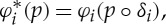
where *δ*_*i*_ is the canonical injection 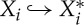 mapping each *j* to the vertex of the simplex at which the *jth* coordinate is 1.

**Definition 5.4 (mixed strategy generalized Nash equilibrium)**

Let 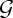 be a finite game. A strategy profile for 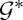 will be called a *mixed strategy profile* for 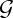. A generalized Nash equilibrium of 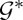 will be called a *mixed strategy generalized Nash equilibrium* of 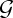.

The most important property of mixed extensions is that they are always multilinear. This will used to prove the generalized Nash theorem.

**Lemma 5.5**

*Let* 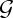 *be a finite game. Then* 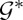 *is a multilinear game*.

**Proof.**

Each *Δ*_*n*_ for *n*>0 is a non-empty, compact and convex subset of the locally convex space 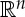. Continuity of the 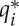 is clear. 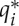 is multilinear because

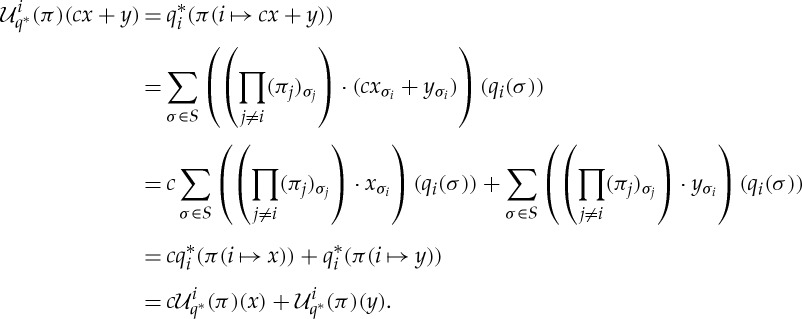

The 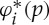 are of the form *φ*_*i*_(*p*′) for some *p*′, and so are closed and convex. We note that 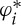 is total because *φ*_*i*_ is. The graph of 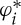 is

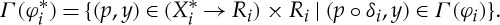
Suppose we have a convergent sequence (*p*_*j*_,*y*_*j*_)→(*p*,*y*) in 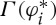. Let *x*∈*X*, then

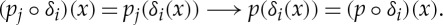
Therefore, *p*_*j*_°*δ*_*i*_→*p*°*δ*_*i*_ pointwise, so

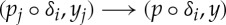
with respect to the topology of pointwise convergence. Because the unilateral topology is finer than the topology of pointwise convergence, we are done. □

The final result we need is the ability to lift selection functions to mixed extensions.

**Lemma 5.6**

*Let X be a non-empty finite set and let* 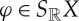 *be a total quantifier attained by the selection function* 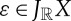. Then there exists a selection function ε* *such that* φ* *is attained by* ε*.

**Proof.**

We define 
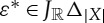
 by the equation

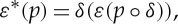
where *δ*:*X*↪*Δ*_|*X*|_. Then

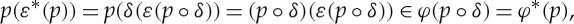
for all 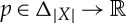. □

**Theorem 5.7 (existence theorem for finite games)**

*Let* 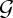 *be a finite game such that each φ*_*i*_ *is attained by a selection function. Then* 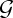 *has a mixed strategy generalized Nash equilibrium.*

**Proof.**

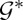
 is a multilinear game which is attained by selection functions by lemmas 5.5 and 5.6. Therefore, 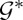 has a generalized Nash equilibrium by theorem 4.13. □

In order to recover the classical Nash theorem, we simply consider finite games whose outcome spaces are 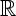 and define 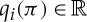 to be the utility of *π* for player *i*, taking all quantifiers to be 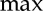. We could instead define a finite game with single outcome space 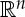 and let (*q*(*π*))_*i*_ be the utility of *π* for player *i*, and consider selection functions 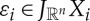 maximizing the *ith* coordinate:

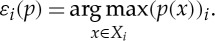
However, the quantifiers attained by these selection functions have closed graph only if *n*=1. This game has the same equilibria as the equivalent game with multiple outcome spaces, but the Nash theorem cannot be proved in this way. It is for this reason that we need to consider games with multiple outcome spaces, in contrast to generalized sequential games (which do not require continuity).

For a different example of a quantifier in a finite game, let *R*_*i*_ be normed and fix *ε*>0 and *x*_0_∈*X*_*i*_. Define

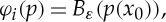
that is, the closed *ε*-ball around *p*(*x*_0_). This quantifier is attained by the constant selection function *ε*(*p*)=*x*_0_. For a sequential game this would force the game to be trivial, but this is not the case here: for example, if *φ*_*X*_ is the quantifier defined here and *φ*_*Y*_ is the maximum quantifier with 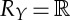 then a Nash equilibrium is a point (*a*,*b*) such that

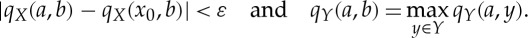
It is hard to find an intuition for a game using this quantifier, but it serves to show that arg max and *arg* *min* are not the only quantifiers satisfying definition 5.1, which in turn shows that the generalization from Nash's theorem to theorem 5.7 is not vacuous.

## 6. The normal form of a sequential game

In classical game theory, every game can be put into the form of a simultaneous game called its *normal form*. The major motivation for defining generalized simultaneous games was to generalize this operation to give a notion of normal form for generalized sequential games. This construction is given in this section and a form of soundness of proved, namely that the solution concept for a generalized sequential game, the so-called *optimal strategies*, are mapped to generalized Nash equilibria.

**Definition 6.1 (generalized sequential game)**

A *generalized sequential game* with *n* rounds is determined by a set *R* of *outcomes*, a set *X*_*i*_ of *moves* and a quantifier *φ*_*i*_∈*S*_*R*_*X*_*i*_, for each 1≤*i*≤*n*, and an *outcome function* 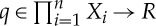. A *strategy* in a sequential game is a tuple

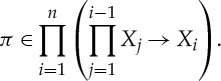
This definition of strategy gives the dynamic structure of a sequential game: now the component *π*_*i*_ of a strategy for the *ith* round of a sequential game is a choice of move for each possible partial play of the game up to round *i*.

A *partial play* of the game is any tuple 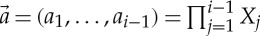, where 1≤*i*≤*n*. Given a strategy *π* and a partial play 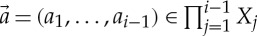, we define the *strategic extension* of 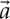 by *π* as

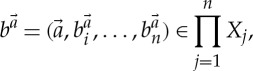
where

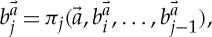
for *i*≤*j*≤*n*. The strategy *π* is called *optimal* iff for all partial plays 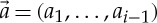 we have

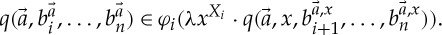

Given a strategy *π* in a game, its *strategic play* 
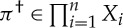
 is given by the strategic extension by *π* of the empty partial play (that is, taking *i*=1 in the definition of a partial play). The strategic play of an optimal strategy is called an *optimal play*.

To be precise, this notion of sequential game is called a *finite game with multiple optimal outcomes* in [1]. Infinite sequential games are avoided in this paper because they are not well-behaved in a classical set-theoretic foundation (see §2*a*).

Generalized sequential games were introduced in order to study a particular higher-order function called the *product of selection functions*. This is the function

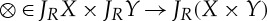
given by

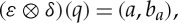
where

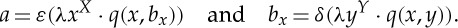
This can be finitely iterated by the recursion

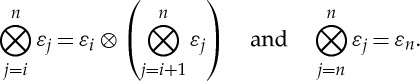
Moreover, in certain foundations (although not in classical set theory), this can be extended to products of countably many selection functions. This infinitely iterated product has many interesting and unintuitive properties: for example, it computes witnesses for the axiom of countable choice, and computes exhaustive searches of certain infinite types in finite time [4], both of which popular belief would have is impossible. Every use of the product of selection functions can be seen as the computation of an optimal play for a suitable generalized sequential game.

**Theorem 6.2**

*Let* 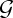 *be a generalized sequential game whose quantifiers are total and attained by selection functions ε*_*i*_*∈J*_*R*_*X*_*i*_*. Then
*
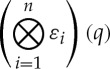
*is an optimal play for* 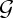
*.*

This result follows from theorem 5.4 of [1], and is stated in the remark following lemma 5.1 of that paper. It should be read analogously to theorem 5.7: both state that every game of a certain kind has a solution. An important difference is that while theorem 5.7 is non-constructive (relying on the non-constructive Kakutani–Fan–Glicksberg theorem), theorem 6.2 gives a way to compute optimal strategies using the product of selection functions.

Now we give the normal form construction and prove that it maps optimal strategies to generalized Nash equilibria. The intuition for this construction is the same as in the classical case: instead of players taking turns choosing moves, rather they simultaneously choose contingent strategies for the entire game. The outcome functions of the game are altered so that they first ‘play out’ the chosen strategy profile to produce a play of the game, which can then be used to produce an outcome.

**Definition 6.3 (normal form)**

Let 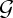 be a generalized sequential game. We define a simultaneous game with single outcome space

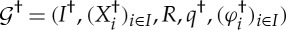
called the *normal form* of 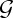 as follows:

—emsp;*I*^†^={1,…,*n*};
—emsp;each 
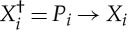
, where 
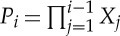
;
—emsp;*q*^†^(*π*)=*q*(*π*^†^) (where *π*^†^ is the strategic play of *π*); and
—emsp;each 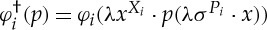.

**Theorem 6.4**

*Let* 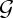 *be a sequential game and let π be an optimal strategy for* 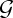
*. Then π is a generalized Nash equilibrium of* 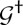
*.*

**Proof.**

Let 1≤*i*≤*n*, where *n* is the number of rounds of 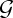. It must be proved that

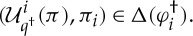

Let 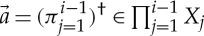. Because *π* is an optimal strategy for 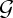, we have

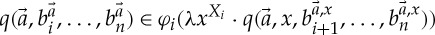
by definition 6.1.

By induction on *j*, we have

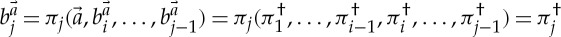
therefore,

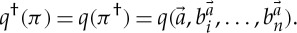

We also have

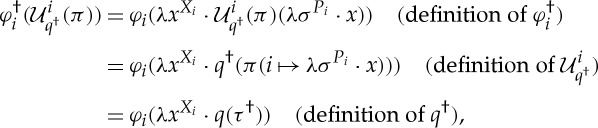
where *τ*=*π*(*i*↦*λσ*^*P*_*i*_^⋅*x*). By induction on *j*, we have

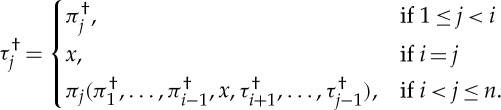
We certainly have that *τ*^†^ coincides with 
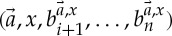
 at indices 1≤*j*≤*i*. Moreover, by induction on *i*<*j*≤*n*, we have

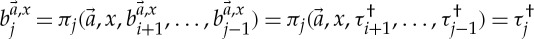
therefore,

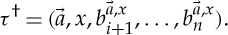

From the assumption that *π* is an optimal strategy of 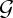 we have, therefore, deduced

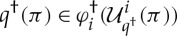
that is,

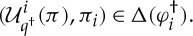
 □

The converse is false because optimal strategies of sequential games generalize the classical notion of subgame-perfect equilibrium, which is a stronger condition than classical Nash equilibrium (called an *equilibrium refinement* in classical game theory; [15]).

## 7. Two-player games and minimax strategies

In this section, the abstract notion of a *ψ*–*φ* strategy is defined, and used to show an intriguing connection between generalized simultaneous games and proof theory. The reason for this terminology is that a *ψ*–*φ* strategy corresponds to a minimax strategy, that is a strategy where each player plays so as to minimize their maximum loss, in a two-player game with 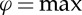 and 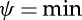. Note, however, that when modelling a classical game as a generalized game, all the quantifiers will be 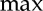, and so *ψ*–*φ* strategies are distinct in this sense from minimax strategies.

**Definition 7.1 (ψ –φ strategy)**

Let 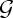 be a two-player game with outcome functions *q*_*X*_∈*X*×*Y* →*R*_*X*_, *q*_*Y*_∈*X*×*Y* →*R*_*Y*_ and quantifiers *φ*∈*S*_*R*_*X*__*X*, *ψ*∈*S*_*R*_*Y*__*Y* . A strategy *a*∈*X* is called a *ψ*–*φ strategy* for player 1 iff

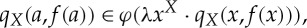
for all *f*∈*X*→*Y* with the property that for all *x*∈*X*,

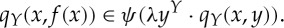
Similarly, *b* is a *ψ*–*φ* strategy for player 2 iff *q*_*Y*_(*g*(*b*),*b*)∈*ψ*(*λy*^*Y*^⋅*q*_*Y*_(*g*(*y*),*y*)) whenever *q*_*X*_(*g*(*y*),*y*)∈*φ*(*λx*^*X*^⋅*q*_*X*_(*x*,*y*)), for all *y*∈*Y* . A *ψ*–*φ strategy profile* is one whose components are both *ψ*–*φ* strategies.

The *binary Berardi–Bezem–Coquand functional* is the higher-order function

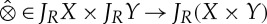
given by

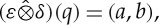
where

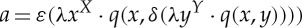
and

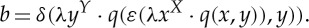
Notice that the type of 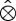 is the same as the type of ⊗. Moreover, when infinitely iterated (again in certain non-classical foundations) both provide proof interpretations (in the modified realizability interpretation of Heyting arithmetic) of the axiom of countable choice [16] and a certain equivalence, namely interdefinability over system T, is shown in [17]. However, the relationship between ⊗ and 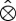 is not well understood, and only ⊗ has previously been linked to game theory.

**Theorem 7.2**

*Let* 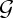 *be a two-player game with single outcome space R, outcome function q ∈ X × Y → R, and total single-valued quantifiers φ, ψ attained by selection functions ε∈J*_*R*_*X, δ∈J*_*R*_*Y . Then the strategy profile
*
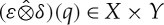
*is a ψ–φ strategy profile.*

**Proof.**

Because *ψ* is single-valued, the unique *f* with the property that

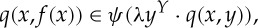
for all *x*∈*X* is

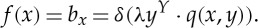
The first component in the strategy profile is

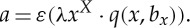
We, therefore, have

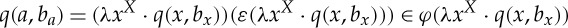
as required. The proof for *b* is symmetric. □

In particular, two-player zero-sum classical games are of this form. In a zero-sum game the outcome is a single real number given by an outcome function 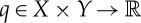, which the first player tries to maximize and the second tries to minimize. The strategy profile given by

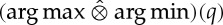
is a minimax strategy, that is, each player's component is chosen to minimize their maximum loss.
